# Parkinson’s disease and occupational exposure to organic solvents in Finland: a nationwide case-control study

**DOI:** 10.5271/sjweh.4125

**Published:** 2024-01-01

**Authors:** Markku Sallmén, Igor Burstyn, Sanni Uuksulainen, Aki Koskinen, Christer Hublin, Markku Sainio

**Affiliations:** † Deceased December 2022; 1 Finnish Institute of Occupational Health, Helsinki, Finland.; 2 Occupational and Environmental Health, Dornsife School of Public Health, Drexel University, Philadelphia, PA, USA.; 3 Outpatient Clinic for Functional Disorders, HUS Helsinki University Hospital, Helsinki, Finland.

**Keywords:** chlorinated hydrocarbon, exposure measurement error, job-exposure matrix, probabilistic bias analysis

## Abstract

**Objective:**

This study aimed to investigate the association between Parkinson’s disease (PD) and occupational exposure to organic solvents generally and chlorinated hydrocarbons (CHC) in particular.

**Methods:**

We assembled a Finland-wide case–control study for birth years 1930–1950 by identifying incident PD cases from the register of Reimbursement of Medical Costs and drawing two controls per case using incidence density sampling from the Population Information System, matched on sex, birth year, and residency in Finland in 1980–2014. Occupation and socioeconomic status (SES) were identified from national censuses. We assessed cumulative occupational exposures via FINJEM job-exposure matrix. Smoking was based on occupation-specific prevalence by sex from national surveys. We estimated confounder-adjusted PD incidence rate ratios (IRR) via logistic regression and evaluated their sensitivity to errors in FINJEM through probabilistic bias analysis (PBA).

**Results:**

Among ever-employed, we identified 17 187 cases (16.0% potentially exposed to CHC) and 35 738 matched controls. Cases were more likely to not smoke and belong to higher SES. Cumulative exposure (CE) to CHC (per 100 ppm-years, 5-year lag) was associated with adjusted IRR 1.235 (95% confidence interval 0.986–1.547), with stronger associations among women and among persons who had more census records. Sensitivity analyses did not reveal notable associations, but stronger effects were seen in the younger birth cohort (1940–1950). PBA produced notably weaker associations, yielding a median IRR 1.097 (95% simulation interval 0.920–1.291) for CHC.

**Conclusion:**

Our findings imply that PD is unlikely to be related to typical occupational solvent exposure in Finland, but excess risk cannot be ruled out in some highly exposed occupations.

Parkinson’s disease (PD) (1) is a multifactorial neurodegenerative disease, characterized by slow movements, resting tremor, muscle rigidity, and impaired balance. PD is caused by a disturbance in the extrapyramidal neural pathways in the brain, due to cell loss in substantia nigra, resulting in a decrease in the brain transmitter dopamine and accumulation of Lewy-bodies in the brain.

Among environmental exposures, smoking tobacco products and caffeine intake are associated with lower risk of PD (2). Some studies indicate greater risk of PD or parkinsonism with exposure to lead (3, 4), copper (5), and pesticides (3, 6, 7), and welding fumes (8) containing manganese (9). Similarly, solvents are suspected (10), especially chlorinated hydrocarbons like trichlorethylene (11–14). However, many of the studies are small and difficult to interpret due to methodological shortcomings (15). Overall, there is paucity of evidence of associations between work-related factors and neurodegenerative diseases for several reasons (16), including difficulty of identifying cases, poor understanding of the pathologic mechanisms, mixtures of exposures of interest in small workplaces, and possibility for long latency. Limitations, such as rarity of exposures of interest and exposure to mixtures, may be overcome by studies in large-scale settings (16).

This nationwide case–control study aims to investigate the association between PD and occupational exposure to organic solvents. Chlorinated hydrocarbons (CHC) are of particular interest due to positive findings in an earlier twin study (11) and mechanistic evidence (10), as well as suggestive results from earlier nationwide study in Finland (17).

## Methods

### Selection of cases and controls

**Figure 1:**
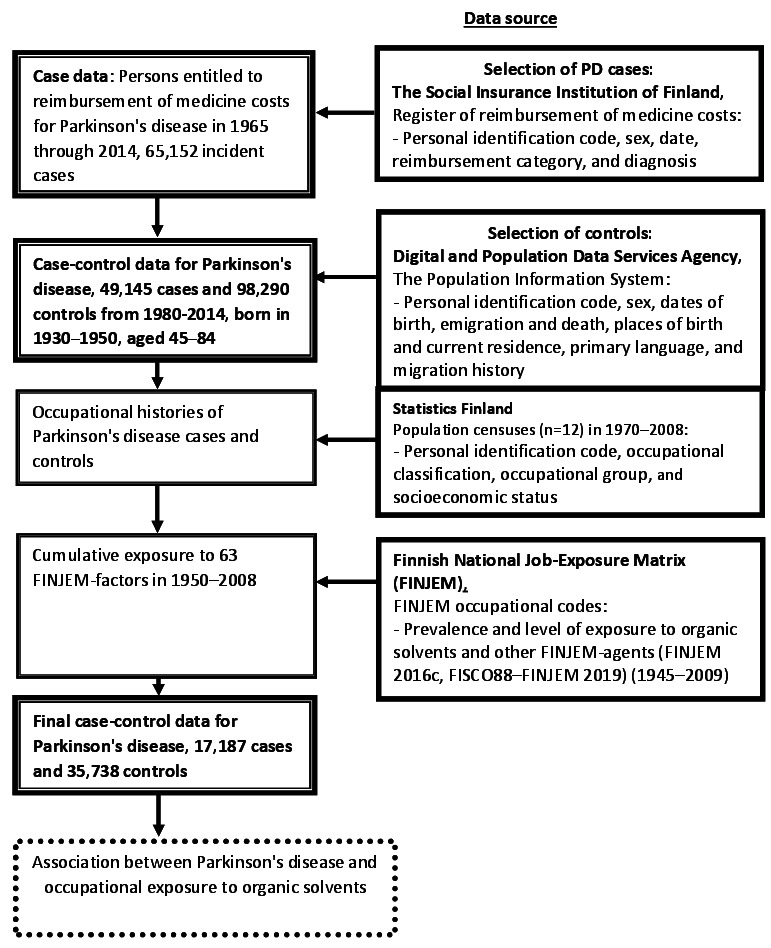
Data generation and data sources for the case–control study of Parkinson's disease.

Ethics approval was not required for this register-based study. PD cases (65 152 persons, first reimbursement) in 1965–2014 were obtained from the register of Reimbursement of Medical Costs for PD (code 110: “PD and comparable movement disorders”) maintained by the Social Insurance Institution of Finland (FSII) (figure 1). Reimbursement is granted after submission of a medical certificate by the treating neurologist and review by medical experts of the FSII. Since 1990, the register included the 9^th^ and 10^th^ revision of the International Classification of Diseases [332 in ICD-9 (1987–1995)], G20 in ICD-10 (since 1996) diagnosis code for some patients, and for all the patients since 2000. We selected as cases persons with reimbursement code 110 either with or without PD diagnosis. In 95% of diagnosed cases, the diagnosis was “Parkinson disease” in the 1990s. Using incidence density sampling, the Population Information System, maintained by the Digital and Population Data Services Agency, selected two potential controls per case having the same sex and birth year, and living in Finland on the case registration date (hereafter “index date”). Age of the controls at index date was within a year of the matching case.

One case was excluded because they elected to not share data for research, resulting in 49 145 cases and 98 290 controls for cases restricted to 1980–2014. The study was restricted to Finnish- or Swedish-speaking subjects (813 excluded) aged 45–84 in the index years (13 062 excluded). PD is uncommon before 45 years of age (18) and genetic susceptibility may dominate over occupational factors at younger age (19). Under-ascertainment of PD becomes more prominent after 85 years of age (20, 21). We further excluded 21 896 subjects due to the following, in part overlapping reasons: (i) 19 847 with no active censuses in 1970–1990 [thus no information on exposure and thereby limiting study to those known to have been ever-employed, smoking or socioeconomic status (SES)]; (ii) 972 born abroad (a heterogeneous group of persons from many countries and not captured by oldest national censuses, and their risk to get PD may differ not only from Finnish residents but also from their own source populations (22); (iii) 839 PD cases with reimbursement for Alzheimer disease before PD index date; and (iv) 790 potential controls who were registered for reimbursement of PD-related costs prior to control selection. The resulting sample included 36 621 cases and 75 041 controls.

Differential matching between census data on occupations and working age by birth year motivated further restriction of cases and controls. Due to the wide range of birth years, working age overlapped poorly with the occupational data (detailed below). Only for 8% of the subjects, occupation could be determined for the entire typical working life. For most of the subjects, the pre-census occupational history (1950–1967) was more than half of the entire working life (supplementary material, www.sjweh.fi/article/4125, table S1), and thus was afflicted with lower quality of information available to estimate exposure than the younger birth cohorts. Consequently, in an approach identical to Nielsen et al (17), we restricted the final study population to those born in 1930–1950 (17 187 cases and 35 738 controls).

### Occupational histories

Occupational history came in the form of occupational codes in the census records that were linked to the Finnish job-exposure matrix called FINJEM. Occupational histories were coded to match FINJEM 2016c (23) or FISCO88-FINJEM 2019 (24). Using 11-digit personal identification code, we linked the case–control data at Statistics Finland to 12 population censuses carried out every five years in 1970–2000 and annually in 2004–2008. Censuses include codes for occupation and SES. Correspondence between population censuses, three classification systems for the occupation codes in theses census, and FINJEM periods are presented in supplementary table S2. Three occupational classifications were used in the available censuses: Classification of Occupations of Statistics Finland’s longitudinal file (LCF) in 1970–1985; Classification of Occupations 1980 (VAL80) in 1990; and Classification of Occupations 2001 (FISCO88) in 1995–2008. All of the 448 VAL80 occupational codes convert directly to a single FINJEM occupational code. However, seven of the 373 LCF occupational codes, representing about 8% of the LCF data, split to two or more FINJEM codes, and 196 of the 445 FISCO88 occupational codes, about 74% of the FISCO88 data, split to two or more FINJEM codes. We addressed splits following the method of Sallmen & Uuksulainen (25). We resolved about 45% of the split LCF codes in 1970–1985 into a single FINJEM code by examining personal occupational histories backwards from the 1990 FINJEM code: a single FINJEM code was accepted for a split LCF code when the 1990 FINJEM code was one of the split candidates. To resolve the split FISCO88 codes into FINJEM codes, we used forward examination of occupational histories between the 1990 FINJEM code and later FISCO88 codes. These solutions and direct FISCO88-FINJEM conversions totaled about 88% of occupational codes in census 1995, but <50% in 2008 due to occupational changes. The remaining split FISCO88 occupational groups are more heterogenous than actual FINJEM occupational groups (hereafter “FINJEM occupation”) and have low prevalence of solvent exposure, meaning that they were assessed as unexposed by FINJEM. In all the classifications, occupational groups include from a few up to 100–150 individual occupational titles.

FINJEM occupation for each year was assumed to be that in the closest census (supplementary table S2). We considered the study subject occupationally inactive if they had no occupational code, occupation was unknown, or were either student, pensioner, or unemployed.

### Occupational exposure assessment

Exposure assessment was blind to PD status. Exposure was assessed for 63 FINJEM agents with exposure estimates in FINJEM period 1945–2009. For each subject, we calculated cumulative exposure (CE) such that each subject’s exposure histories were complete and fell within the years 1950–2008, following imputation of annual exposure in the pre-census years that accounted for a pronounced increase in industrialization over time (supplementary table S3). CE for each FINJEM agent was based on the person’s FINJEM occupation in each census, activity at census year, and FINJEM estimates for 1945–2009 (supplementary table S2) in job exposure matrix (FINJEM 2016c or FISCO88–FINJEM 2019). In each calendar year, annual mean exposure for each agent was the product of activity (0/1), the annual backward stability coefficient (supplementary table S3, range 0–1 for the pre-census years 1950–1967 and value 1 for 1968–2008; based on the judgement of the authors), the probability (P) and the average level (L; for solvents as ppm in the air) of exposure in a given FINJEM occupation and year. To assess CE for solvents as ppm-years, we summed the annual exposure estimates from years the subject was 20–65 years until index (diagnosis or selection) year minus six years (minimum five-year lag). We applied a minimum lag of five years because motor symptoms of PD are present throughout this period (1), in particular to such an extent that it is possible that these symptoms, for example leading to injurious falls (2, 4), could impact the ability to work or conduct some tasks during this time. Risk estimates were made per 100 ppm-years in order to make them relevant to typical exposures in Finland in 1960–1984, such that this level of accumulated exposure to chlorinated solvents would be reached by (a) typical persons in 5.5 years due to median level of exposure of 18 ppm and (b) the most exposed persons in 2 years due to maximum level of exposure of 50 ppm.

### Covariate assessment

SES (five categories: self-employed farmers, other self-employed entrepreneurs, upper-level employees, lower-level employees, manual workers) was defined from the last active census in 1970–1990. FINJEM occupation- and sex-specific regular smoking prevalence (hereafter, smoking prevalence) was derived from the annual health surveys in 1978–1991 (prevalence estimates years) (26). Smoking prevalence for each subject was assessed from the FINJEM occupations during survey period (census order 1985, 1980, and 1990) and, if missing, based on FINJEM occupations in 1975 and 1970. For rare FINJEM occupations without smoking data, prevalence was assumed to be that of closely related larger occupations.

Because of the wide range of birth years (1896–1969), we extended the occupational history to the years 1950–1967 using the earliest available census record. In Finland, there was a pronounced industrialization after World War II. Consequently, the industrial FINJEM occupation workforce in FINJEM period 1945–1959 was about half of that in 1960–1984 (23). To address this, we created annual FINJEM occupation-specific backward stability coefficients for the pre-census years 1950–1967 (see supplementary table S3). In assessing annual exposure in pre-census years 1950–1967, FINJEM prevalence of exposure was multiplied by annual occupation-specific backward stability coefficients (range 0–1). In line with industrialization, a monotonic increase in prevalence of solvent exposure from 1950–1967 was assumed for most solvent-exposed occupations. However, in about 40 FINJEM occupations the number of workers declined (eg, agricultural occupations), and stability of 1 was used for all years 1950–1967.

### Statistical analyses

*Conventional analyses*. We used SAS version 9.4 (SAS Institute, Inc, Cary, NC, USA) to analyze associations between CE to organic solvents and PD. We used unconditional logistic regression to estimate incidence rate ratios (IRR) and their 95% confidence intervals (Cs) (27). To control for matching (28), we adjusted for sex and birth year, and forced continuous smoking prevalence and categorical SES into the multivariable model.

In categorical analyses, the reference category for solvents and other FINJEM agents included persons unexposed in all the calendar years used to compute CE. Based on numbers of potentially exposed subject, two to four categories of exposure were defined for all the FINJEM agents with 10–20% of the exposed allocated to the highest category.

We had few *a priori* confounders among occupational factors. However, there are reasons to suspect that welding (in part due to manganese and other metals) is implicated in PD (9). Therefore, we forced welding and chromium (strong correlate of nickel; manganese not assessed in FINJEM due to its rarity in Finland) into multivariable models. Likewise, correlated aliphatic/alicyclic hydrocarbon solvents and aromatic hydrocarbon solvents (Pearson coefficient 0.76) were individually evaluated in multivariable models to check for potential confounding. We retained potential occupational confounders in final analyses if they altered IRR by 10% or more, as recommended by Maldonado & Greenland (29), while noting that this should not be confused with definitive identification of any covariate as true confounder (30). We conducted analyses stratified by sex, birth cohort, and the continuity of the same occupation (as number of successive economically active censuses).

### Probabilistic bias analysis (PBA) to account for elements of exposure measurement error

Following conventional analysis, we evaluated the extent of bias that the use of FINJEM to estimate cumulative occupational exposures may have induced in the conventional analysis. Specifically, we focus on bias due to estimation of occupational exposures for an occupation as a product of probability (P) (modified by the backward stability coefficient where appropriate) and arithmetic mean (L). In doing so, we followed the approach of PBA (31, 32). We utilized the exposure model developed by Burstyn et al (33) and default prior on variance of occupational exposures given in Method II of Jones & Burstyn (34). Technical details and SAS implementation are presented in supplementary appendix 1; heuristic overview follows. We replaced each annual exposure estimate (the P×L) derived from linkage of FINJEM and census occupational records for each participant with values that reflect the chance that either (i) the subject was truly unexposed, in which case zero was imputed (namely, if P=0, we kept the original unexposed status, but if P>0, then a proportion of subjects equal to (1-P) were imputed as unexposed) or (ii) the subject was truly exposed, in which case an exposure from a lognormal distribution with arithmetic mean L and simulated variance was imputed for the P proportion of subjects in a given occupation. The associations of CE and PD were estimated as in the conventional analysis for each set of imputed exposures, with the following caveat. We randomly sampled IRR from the distribution defined by its point estimate and standard error. We summarized the distribution of these sampled values, which reflect adjustment for bias due to both systematic errors (including aggregation bias due to FINJEM) and random errors across imputations, as a simulation interval (SI), in terms of its observed median, 2.5^th^ and 97.5^th^ percentiles. We achieved stability in the estimated medians of the IRR after 400 sets of imputed exposures.

We also considered in a separate PBA that individuals who held the same occupation in consecutive two to five censuses 1970 through 1990 may have had the same chance of being exposed during the entire period, not varying from year-to-year, referred hereafter as “the stability constraint”. Specifically, we simulated probability of having been exposed during such years of occupational stability based on FINJEM assigned value of P arising from linkage to the first census record. For example, for subjects with the same occupation in five censuses between 1970 and 1990, if the subject was simulated as having been exposed in 1970, they were classified as exposed throughout 1968 to 1992 and unexposed otherwise. Exposure intensity in the exposed years was derived from year-specific values of L as above. The rest of PBA analysis proceeded as described above.

## Results

Characteristics of PD cases and controls are presented in table 1. The distributions of matching variables, birth year and sex, were similar in cases and controls. Proportion of women reduced from 49% (original population) to 42% (final study population) due to higher total occupational inactivity in censuses 1970–1990 compared to men (23.3% versus 6.8%). There was an inverse association with SES and PD. More controls smoked than cases. Mean age of cases at index date was 66.38 years, range 44.11–84.68 and in controls 66.50 years, range 44.01–84.69.

**Table 1:** Characteristics of Parkinson’s disease cases and controls, Finland 1980–2014.

Factor		Cases (N=17 187)	Controls (N=35 738)
		%	%
Women		41.8	42.0
Year of birth			
	1930–1939	62.8	63.6
	1940–1950	37.3	36.4
Socioeconomic status			
	Self-employed farmers	12.5	11.5
	Other self-employed entrepreneurs	7.4	8.0
	Upper-level employees	15.5	14.2
	Lower-level employees	27.5	27.0
	Manual workers	37.1	39.4
Prevalence of regular smokers ^a^, men			
	5–14	0.7	0.6
	15–24	26.0	23.0
	25–34	28.2	27.1
	35–44I	37.7	40.7
	45–62	7.4	8.6
Prevalence of regular smokers ^a^, women			
	0–4	6.2	6.8
	5–14	29.2	26.9
	15–24	51.0	52.9
	25–34	11.2	11.8
	35–46	2.4	2.6

^a^ Prevalence (probability) was based on occupation-specific prevalence of regular smoking for each sex derived from annual health surveys (see text for details); these were divided into 5 rubrics for presentation, with different ranges for men and women.

The associations of PD and categorical CE to organic solvents are shown in table 2. A majority of cases and controls had no exposure to each organic solvent (Table 2), with 16.0% of cases ever potentially exposed to chlorinated hydrocarbon (CHC) solvents. We observed that exposure to any CHC solvent at a level of 20 to 300 ppm-years (5-year lag) was associated on average with an increased risk (adjusted IRR 1.09, 95% CI 0.98–1.21). Among individual solvents, increased adjusted IRR were observed in the highest exposure categories of 1,1,1-trichloroethane (1.16, 95% CI 1.02–1.31) and methylene chloride (1.20, 95% CI 1.03–1.39). There was no evidence of association of PD with exposure to other solvents.

**Table 2:** Parkinson’s disease and cumulative exposure (CE) to organic solvents (ppm-years) among residents of Finland born in 1930–1950. Incidence rate ratios (IRR) and 95% confidence intervals (CI) from logistic regression analysis, adjusted for sex, birth year, socioeconomic status, and occupation- and sex-specific prevalence (probability) of smoking regularly. % refers to proportion of cases in each category.

FINJEM agent		CE (PPM-years)	Cases N (%) ^a^	IRR	95% CI
Chlorinated hydrocarbons					
	Any	0	14 439 (32.6)	1.0	Ref.
		>0–4.9	1 252 (31.3)	0.96	0.89–1.03
		5–19	916 (31.4)	0.98	0.91–1.07
		20–300	580 (33.2)	1.09	0.98–1.21
	Methylene chloride	0	15 989 (32.5)	1.0	Ref.
		>0–4.9	50 (31.3)	0.98	0.89–1.09
		10–14.9	391 (30.4)	0.95	0.84–1.07
		15–90	267 (35.1)	1.20	1.03–1.39
	Perchloroethylene	0	16 845 (32.5)	1.0	Ref.
		0–4.9	219 (31.0)	0.96	0.82–1.13
		5–145	123 (32.0)	1.03	0.83–1.28
	1,1,1-trichloroethane	0	15 534 (32.6)	1.0	Ref.
		>0–3.9	872 (30.5)	0.94	0.87–1.02
		4–9.9	387 (30.4)	0.95	0.84–1.08
		10–75	394 (34.4)	1.16	1.02–1.31
	Trichloroethylene	0	15 816 (32.6)	1.0	Ref.
		>0–4.9	617 (30.9)	0.95	0.86–1.05
		5–14.9	416 (31.2)	0.97	0.87–1.10
		15–225	338 (32.0)	1.03	0.90–1.18
	Aliphatic/alicyclic hydrocarbons	0	15 243 (32.6)	1.0	Ref.
		>0–4.9	1 060 (30.9)	0.96	0.89–1.04
		5–39	525 (31.7)	1.00	0.90–1.12
		40–560	359 (31.2)	0.99	0.87–1.13
Aromatic hydrocarbons					
					
	Any	0	12 174 (32.9)	1.0	Ref.
		>0–2.9	3 753 (31.7)	0.99	0.95–1.05
		3–74.9	815 (30.5)	0.95	0.87–1.03
		75–1080	445 (32.1)	1.04	0.92–1.16
	Benzene	0	15 762 (32.5)	1.0	Ref.
		>0–1.9	1094 (32.4)	1.02	0.95–1.11
		2–90	331 (31.8)	1.03	0.90–1.18
	Styrene	0	14 899 (32.6)	1.0	Ref.
		>0–0.4	1 382 (31.8)	1.01	0.95–1.09
		0.5–4.9	763 (32.2)	1.03	0.94–1.13
		5–685	143 (31.0)	1.00	0.82–1.22
	Toluene	0	15 884 (32.6)	1.0	Ref.
		>0–19	721 (31.2)	0.96	0.88–1.05
		20–99	350 (32.0)	1.03	0.90–1.17
		100–600	232 (31.0)	0.99	0.84–1.15

^a^ Number of Parkinson’s disease cases in the respective exposure category, and percent of the subjects in the respective exposure category who are cases, based on 17 187 cases and 35 738 controls.

These associations by exposure categories were reduced once we considered measurement error in exposure via PBA without the stability constraint. Specifically, the estimated medians of IRR with the highest categories became 1.06 [95% simulation interval (SI) 0.95–1.21], 1.04 (95% SI 0.87–1.24), and 1.03 (95% SI 0.86–1.26), for CHC, 1,1,1-trichloroethane, and methylene chloride, respectively.

Except in trichloroethane, we did not observe a change in IRR close to 10% upon adjustment for *a priori* selected potential confounder. Details of these analyses are shown in supplementary table S4. Therefore, specific occupational confounders were not considered further in our work.

Conventional CHC analyses are summarized in table 3 using continuous cumulative exposure variable for chlorinated hydrocarbons. They indicated that CE to CHC (per 100 ppm-years, 5-year lag) was associated with increased risk (IRR 1.237; 95% CI 0.987–1.550), after accounting for matching variables and SES (IRR 0.987 (95% CI 0.919–1.060), 0.904 (95% CI 0.830–0.985), 0.977 (95% CI 0.918–1.041), and 0.925 (95% CI 0.867–0.986) for SES 1, 2, 4, and 5 compared to 3, respectively), and smoking (0.546; 95% CI 0.420–0.709). Effect estimates were stronger among women than men, and among persons who were economically active in a greater number of censuses, most notably among those active in all five censuses 1970–1990: CHC-IRR 1.526, 95% CI 1.139–2.045.

**Table 3:** Parkinson’s disease case-control study: analyses of continuous chlorinated hydrocarbon cumulative exposure (in 100 ppm-years; 5-year lag) using conventional approach and after probabilistic bias analysis (PBA); [IRR=incidence rate ratios estimated from odds ratios under incidence density sampling; CI=confidence interval; SES=socioeconomic status; SI=simulation interval].

Strata		Covariates in multivariable model	Cases	Controls	Conventional			PBA	
					IRR	95% CI		IRR	95% SI
All subjects		Birth year and sex	17 187	35 738	1.064	0.851– -1.330			
		Additionally: SES and smoking			1.237	0.987– -1.550		1.097	0.920– -1.291
Sex									
	Men	Birth year, SES, smoking	10 010	20 715	1.200	0.900– -1.601		1.079	0.839–1.315
	Women	Birth year, SES, smoking	7 177	15 023	1.287	0.897– -1.847		1.185	0.843–1.651
Birth year									
	1930–1939	Sex, SES, smoking	10 785	22 737	1.098	0.852– -1.416		1.047	0.828–1.281
	1940–1950	Sex, SES, smoking	6 402	13 001	2.007	1.214– -3.319		1.226	0.868–1.811
Active censuses in 1970–1990									
	≥3	Sex, SES, smoking	15 412	32 446	1.327	1.053–1.672		1.113	0.910–1.342
	≥4	Sex, SES, smoking	13 358	28 110	1.313	1.027– -1.678		1.107	0.902– -1.364
	5	Sex, SES, smoking	8 870	18 993	1.526	1.139– -2.045		1.169	0.927– -1.500

PBA finding that accounted for exposure measurement error are summarized in table 3, alongside with the corresponding conventional analyses. They showed weaker association for CHC, suggesting that the conventional estimates may have over-estimated IRR. Specifically, association with CE of CHC (100 ppm-years, 5-year lag) reduced to median IRR of 1.097 (95% SI 0.920–1.291). Accounting for stability of occupational histories further reduced the estimate, with median IRR of 1.040 (95% SI 0.904–1.187). There was a noticeable impact effect of PBA among the most economically active persons in 1970–1990, with median IRR reducing to 1.169, 95% SI 0.927–1.500.

In conventional analysis, cumulative CHC exposure by 1930–1939 birth cohort shows lower IRR relative to the younger birth cohort (1940–1950), which had adjusted IRR 2.007 (95%CI 1.214–3.319) (table 3). This estimate for the 1940–1950 birth cohort reduced in PBA to the median IRR of 1.226 (95% SI 0.868–1.811) (table 3). We tested heterogeneity of IRR between birth cohorts after PBA via a Wald-type test and obtained P=0.15, implying that evidence for effects truly differing by birth cohort is weak. Effect estimate become weaker when occupational stability was assumed: IRR 1.090 (95% SI 0.916–1.285).

## Discussion

This nation-wide case–control study from Finland found no clear association between occupational exposure to solvents and PD. Conventional analyses, using FINJEM exposure estimates, suggested positive association for CHC solvents but these findings were in large part related to aggregation bias (33). We observed the well documented inverse association of PD risk and smoking (35) and reproduced the positive association of PD and upper SES (36).

The current case–control study and the study based on probability of exposure (P) in a single-census by Nielsen et al (17) used identical birth cohorts 1930-1950, but not identical participants. Our findings were less sensitive than earlier work to adjustment for other FINJEM factors. Potential for collider-related bias of unexpected direction was higher in single-census study of Nielsen et al (17), and current approach addressed this.

The risk estimates from the study of twins (11) are higher than those we observed. They are subject to several sources of bias and should be interpreted with caution. The exposure assessment method was inferior to FINJEM in that it was not anchored in measurements and has unknown misclassification rates. Given that the exposure assessment employs a permissive definition of exposed as “at least 2% of work time or 1 hour per week” of either hobbies or jobs, and low statistical power, false positives are highly likely (37, 38). The control for genetic confounding may be incomplete because results for monozygotic twins are not presented, presumably because there are too few for informative analysis. Our results are not compatible with a six-fold increase in risk for any ever-exposure to CHC.

The strength of our work lies in its large nation-wide sample, with few missing data, and in the quality of information on smoking that varied by sex and occupation, as well as accounting quantitatively for impact of imperfection in exposure assessment, and mitigation through study design of bias due to outcome misclassification (ie, by limiting the source cohort to years with records of PD diagnosis). The importance of our findings must be framed within consideration of confounding, measurement error and selection biases that may have affected our estimates.

It is difficult for us to undertake a detailed examination of potential selection bias (related to both exposures and the outcome) with respect to both outcomes and exposures because in case–control design we did not access exposure for the entire parent cohort. Such selection bias is always a concern and therefore our conclusions, strictly speaking, only apply to native-born residents of Finland from specific era and age, who were economically active (had census record) and had unambiguous diagnosis. Bias is unlikely to have arisen from incidence density sampling of controls.

We addressed potential confounding by other occupational exposures through adjustment for estimates exposures to metals and solvents. However, the effect estimates with CHC did not alter enough to impact interpretation of the results, with changes of the IRR under 10%. These adjustments for confounding may not have been complete, because there are no well-established risk factors for PD. Nonetheless, we did control for smoking, one of the few established risk factors that varied by occupation and sex. If observed and latent confounding are of similar magnitude (as is often argued, eg (39), the residual confounding in our analysis would cause bias of at most 10%.

Misclassification of outcome can distort exposure-response associations. Censoring identification of cases to under the age of 85 years helped to address the concern about under-diagnosis (17). We were aware of under-representation of people over 80 years of age among PD cases in the FSII register before 2010 and therefore restricted the study to birth cohorts 1930–1950, who were ≤80 years in 2010. In another study using FSII reimbursement register to identify PD cases, it was concluded that different sources of bias are expected to cause some upward bias in the incidence and prevalence figures (40), but we have no reason to believe that such bias would be related to solvent exposure, thereby biasing our evaluation of occupation etiology of PD. Moreover, PD data of the reimbursement register includes perhaps 5% non-PD cases in the 1980s and gradually fewer towards the late 1990s when increasing number of cases had exact diagnosis, and none thereafter. Those born in 1945 were 55 years old in 2000, when diagnostic data were no longer lacking. However, misclassification is more likely for those born in 1930’s and getting PD at a younger age.

Misclassification and errors in exposure estimates may have biased our results in a difficult-to-predict manner, even though exposure assessment was blinded to outcome ascertainment. Errors in exposure can arise in our work from both inaccuracies in occupational histories and exposure assigned on the basis of occupational histories. We will deal with the issue of errors in exposure estimates first.

We addressed concern of exposure measurement error within occupational groups and the resulting aggregation bias through PBA. Our results indicates that, in line with theory in Burstyn et al (33), the conventional estimates using continuous measure of CE are expected to be positively biased. This bias arises in conventional analysis because in a group assessed as exposed “on average” there are (a) many unexposed persons whose exposure is over-estimated, (b) a few exposed persons whose exposure is under-estimated. This has the consequence of shrinking assessed range of exposure relative to the true one, creating a tendency to over-estimate exposure-response slope [see figure 2 in Burstyn et al (33)]. However, there are many other factors at play, such as proportion of truly exposed in a study sample (33). We think that one of the most critical issues here is the low prevalence of solvent exposure. For CHC, the mode and median assigned probability of exposure per occupation was about 5%. For trichloroethylene, 85% of all the potentially exposed were employed in occupations with such low prevalence. Moreover, our decision to extend the exposure assessment into pre-census years 1950–1967 and the use of backward stability coefficients lead to very low prevalence towards 1950 in most of the industrial exposing occupations. We undertook PBA to quantify the impact of the aggregation bias simulating possible true exposure distributions from observed data. If our models and assumptions are correct, one can be certain that the simulations are closer to the true value of IRR than the conventional analyses. To summarize, confidence intervals of conventional analyses do not have 95% coverage of effect estimates consistent with data and assumed models, when error in exposure is ignored. However, it is important to note that uncertainty bounds between conventional and PBA analyses overlap, such that an argument can be made that both analyses are congruent to some degree.

In conventional analyses, we assumed that occupation and exposure was stable in census year plus or minus two years and were identical across census records within a FINJEM period and similar between FINJEM periods. We used two approaches in PBA: standard and stable. In standard PBA approach, exposure level was imputed independently in each calendar year even with identical occupations within person. In stable PBA, we assumed that probability of exposure was the same across consecutive census records 1970–1990 within the same occupation for a participant. In the case of PBA analyses of 1940–1950 birth cohort, this proved to be an important source of uncertainty that can account fully for suggested positive findings with CE to CHC. We do not know where the truth lies between the extremes of stable work and work changing from census to census.

The backward stability coefficients were used to extrapolate exposure prevalence to time periods not covered by censuses when a rapid industrialization took place in Finland after World War II. The use of backward occupational stability coefficients helped us to get more realistic occupational histories by assuming that the likelihood of the census 1970 occupation is gradually decreased towards 1950. Because of small backwards stability coefficients in many industrial occupations, the coefficient-weighted probabilities for CHC solvents became very small towards 1950s. Backward stability was applied as a group-level factor and therefore cannot account for inter-individual variability, thereby only partially addressing the problem of missing information on exposure during pre-census period. These coefficients as based on expert judgement and as such they are one of the limitations of the work. We did consider knowledge of the timing of the introduction of solvents such that we did not extrapolate use of solvent back to the time when it was not available for industrial use. Though we restricted the final study population to subjects born in 1930–1950, the findings may still be more biased negatively in the oldest birth cohorts due to greater uncertainty in exposure assessment than in the younger birth cohorts (this can be further aggravated, as discussed above, by greater outcome misclassification in the older birth cohorts). The patterns evident in both the conventional analysis and PBA is consistent with this idea, if CHC is associated with PD.

A healthy worker survivor effect means tendency of healthier persons to remain employed resulting to higher exposure estimates. In the 1930–1950 birth cohorts, very few subjects supposedly have passed away before the start of follow-up in 1980 but leaving highly exposed jobs is more likely, implying a negative bias. However, in 1930-1940 birth cohort premature deaths may have caused negative bias because heavy solvent exposure is known to cause eg, chronic solvent encephalopathy. Limiting cases to those aged less than 85 years and stratifying analyses by sex, birth cohorts and having been economically active helps alleviate these concerns. However, accessing having been economically active can be confounded by birth cohort.

### Concluding remarks

We conclude that association of PD among persons born 1930–1950 in Finland is unlikely to be attributed to their occupational exposure to solvents, but neither the residual bias, nor excess risk at the extremes of exposure distribution can be ruled out. The suggestion of a stronger effect in a younger cohort with better data may serve as a motivation for future research due to greater relevance of these exposure to current working conditions in Finland. While our results do not exclude the existence of the causal link, current evidence does not support identification of chlorinated solvents as a major preventable cause of PD in Finland.

## Supplementary material

Supplementary file 1
