# Supplementary material for: Parkinson’s disease and occupational exposure to organic solvents in Finland: a nationwide case-control study
Source: Scand J Work Environ Health. 2023 Dec 18;50(1):39–48. doi: 10.5271/sjweh.4125 (PMC10924827; doi:10.5271/sjweh.4125)
Supplement: Supplementary file 1 [file SJWEH-50-39-S001.pdf]

Parkinson's disease and occupational exposure to organic solvents in Finland: a nationwide case-control study[1]

by Markku Sallmén, PhD, Igor Burstyn, MSc, PhD, Sanni Uuksulainen, MSc, Aki Koskinen, MSc, Christer Hublin, MD, PhD, Markku Sainio, MD [2]

1. Supplementary material
2. Correspondence to: Markku Sanio, Outpatient Clinic for Functional Disorders, HUS Helsinki University Hospital, Helsinki, Finland. [E-mail:markku.sainio@hus.fi].

Table S1. Factors related to study quality by birth year: Proportion of pre-census years (PPCY), years using FISCO88 occupational classification of total years assessed for cumulative exposure, and mean age in the first (1980) and the last year (2014) of Parkinson disease (PD) registration.

		**Proportion of pre-census years 1950-1967 in exposure assessment**		**Proportion of FISCO88 occupational code period 1993-2008 in exposure assessment**		**Age at first and last PD index year**	
Birth year	Cases/ controls	Mean PPCY	Range	Mean	Range	Range in 1980	Range in 2014
1891 – 1914	3609/7156	0.74	0.64 – 1.0 *	0		66 – 89 *	100 –
1915 – 1919	3234/6574	0.59	0.55 – 0.72 *	0		61 – 65 *	95 – 99
1920 – 1924	4787/9622	0.52	0.47 – 0.72 *	0		56 – 60 *	90 – 94
1925 – 1929	5896/12 198	0.47	0.42 – 0.72 *	0		51 – 55	85 – 89
1930 – 1934	5884/12 470	0.40	0.32 – 0.72 *	0.04	0.0 – 0.11	46 – 50	80 – 84
1935 – 1939	4901/10 267	0.29	0.20 – 0.65	0.12	0.0 – 0.23	41 – 45	75 – 80
1940 – 1944	3388/6926	0.17	0.09 – 0.40	0.19	0.0 – 0.34	36 – 40	70 – 74
1945 – 1949	2649/5325	0.04	0 – 0.15	0.25	0.0 – 0.40	31 – 35	65 – 70 (*)
1950 – 1954	1369/2754	0	0	0.32 *	0.0 – 0.46	26 – 30	60 – 64 *
1955 – 1959	610/1195	0	0	0.41 *	0.1 – 0.53	21 – 25	55 – 59 *
1960 – 1968	295/561	0	0	0.55 *	0.35 – 0.76	16 – 20	50 – 54 *

Symbols: * indicates poor quality, (*) indicates slightly reduced quality

Zero values in FISCO88 proportion for birth cohort 1945 – 1949, for example, are possible for younger cases and their controls in index years 1998 or earlier with last cumulative exposure year before 1993.

Table S2. Correspondence between population censuses, classification of occupations and FINJEM-periods.

**Census (N=12) (coverage period)**	**FINJEM-period (N=8)**								
	1945–1959	1960–1984	1985–1994		1995–1997	1998–2000	2001–2003	2004–2006	2007–2009
(1950–1967) [d]	X	X							
1970 (1968–1972)		X							
1975 (1973–1977)		X							
1980 (1978–1982)		X							
1985 (1983–1987)		X	X						
1990 (1988–1992)				X					
1995 (1993–1997)				X	X				
2000 (1998–2002)						X	X		
2004 (2003–2004)							X	X	
2005 (2005)								X	
2006 (2006)								X	
2007 (2007)									X
2008 (2008)									X
	LCF[a]			VAL80[b]	FISCO88[c]				
	**Classification of occupations (N=3)**								

a LCF = Classification of occupations of Statistics Finland's longitudinal data for population censuses 1970, 1975, 1980 and 1985 affecting exposure estimates in a 28-year period 1950–1987.

b VAL80 = Classification of occupations 1980 was used in census 1990 affecting exposure estimates in five years 1988–1992.

c FISCO88 = Classification of occupations 2001 was used in seven censuses 1995, 2000, and 2004–2008 affecting exposure estimates in a 16-year period 1993–2008.

d Between 1950 and 1967, there were no censuses. Occupational group assumed to be the one in census 1970. The annual exposure estimates are values in the FINJEM-periods 1945–1959 and 1960–1984 multiplied by backward occupational stability coefficients. See Supplemental table 3.

Table S3: Backwards occupational stability coefficients for historical reconstruction of exposures in pre-census years 1950-1967, and prevalence of CHC exposure (P-CHC) in 1970 and 1950. P-CHC in 1970 directly from FINJEM. P-CHC in 1950 estimated as P-CHC in FINJEM period 1945-1959 multiplied by occupation-specific backward stability coefficient.

CHC exposed occupational groups and respective code	N in census 1970[a]	Backward stability coefficient	Prevalence of CHC exposure in 1970/1950
657 Assemblers and other machine and metalware occupations	248	0.0	0.055/0
702 Lithographers	54	0.1	0.053/0.005
028 Laboratory assistants	175	0.2	0.055/0.016
652 Machine and engine mechanics	694	0.2	0.079/0.02
032-034 Nurses, not elsewhere classified	266	0.3	0.0027/0.00249
663 Electronics and telecommunications workmen	86	0.3	0.1/0.03
665 Electrical and electronic equipment assemblers	94	0.3	0.1/0.03
701 Printers	97	0.3	0.203/0.03
032 Nurses	313	0.4	0.003/0.004
650 Turners, toolmakers, and machine-tool setters	523	0.5	0.055/0.05
680 Painters, lacquerers, and floor layers	384	0.6	0.103/0.072
709 Occupation in graphics, not elsewhere classified	27	0.6	0.068/0.03
752 Plastic product workers	117	0.6	0.05/0.00522
760 Packers and labelers etc.	395	0.6	0.0005/0.0005
613 Upholsterers	44	0.7	0.08/ 0
703 Bookbinders	84	0.7	0.0094/ NA
850 Laundry workers	71	0.7	0.39/0.28
730 Distillers	4	0.8	0.098/ NA
731 Cookers and furnacemen (chemical processes)	18	0.8	0.05/0.04
736 Refinery workers, other occupations in the chemical industry	111	0.8	0.048/0.0152
751 Rubber products workers	78	0.8	0.1/0.04
605 Textile finishers, dyers	62	0.85	0.029/0.0425
600 Fiber processors	10	1.0	0.033/0.05
623 Lasters and sole fitters etc.	6	1.0	0.252/0.5
639 Occupations in smelting, metallurgical and foundry work, not elsewhere classified	62	1.0	0.055/0.1
656 Metal plating and coating work	18	1.0	0.916/0.9
677 Woodworking machine operators etc.	143	1.0	0.013/ 0

[a] Total number of cases and controls of all cases and controls active in the 1970 census.

NA = No subjects of working age in 1950. Zero values in 1950 are P-CHC estimates of no exposure in FINJEM period 1945–1959. Even after considering backward stability coefficients, in some occupations P-CHC was higher in 1950 than in 1970 because of higher P-CHC in FINJEM period 1945-1959 than in 1960-1984.

Table S4. Parkinson's disease and cumulative exposure (CE, as 100 ppm-years) to chlorinated hydrocarbon (CHC) solvents among residents of Finland born in 1930–1950: impact of adjustment for selected FINJEM factors. Incidence Rate Ratios (IRR) and 95% confidence intervals (CI) from logistic regression analysis, adjusted for sex, birth year, socioeconomic status, and occupation- and sex specific prevalence (probability) of smoking regularly. Additionally adjusted for one of the following factors at a time: chromium (CR), nickel (NI), welding (WELD), polycyclic aromatic hydrocarbons (PAH), aliphatic/alicyclic hydrocarbons (ALHC) or aromatic hydrocarbons (ARHC).

**Studied CHC solvent**	**Additional adjustment**	**CHC-IRR**	**95% CI**	**Change in CHC-IRR (%)**
CHC all	Basic model	1.237	0.987–1.550	-
	+ CR	1.258	0.993–1.594	1.7
	+ NI	1.244	0.985–1.558	0.6
	+ welding	1.232	0.983–1.544	-0.4
	+ PAH	1.234	0.984–1.546	-0.2
	+ ALHC	1.302	1.011–1.678	5.3
	+ ARHC	1.278	0.996–1.636	3.3
1,1,1-trichloroethane	Basic model	1.929	0.970–3.836	–
	+ CR	2.085	1.003–4.333	8.1
	+ NI	1.974	0.986–3.854	2.3
	+ welding	1.921	0.966–3.820	-0.0
	+ PAH	1.927	0.969–3.832	-0.1
	+ ALHC	2.384	1.073–5.294	**23.6**
	+ ARHC	2.126	1.002–4.512	**10.2**
Trichloroethylene	Basic model	1.146	0.758–1.733	–
	+ CR	1.162	0.740–1.825	1.4
	+ NI	1.160	0.759–1.773	1.2
	+ welding	1.142	0.755–1.726	-0.3
	+ PAH	1.143	0.756–1.728	-0.3
	+ ALHC	1.142	0.755–1.728	-0.3
	+ ARHC	1.142	0.755–1.726	-0.3
Methylene chloride				

	Basic model	1.591	0.979–2.586	–
	+ CR	1.585	0.973–2.581	-0.4
	+ NI	1.582	0.973–2.571	-0.6
	+ welding	1.574	0.968–2.559	-1.1
	+ PAH	1.581	0.972–2.571	-0.6
	+ ALHC	2.886	1.342–6.207	**81.4**
	+ ARHC	2.393	1.185–4.836	**50.4**
Perchlorethylene	Basic model	1.537	0.946–2.496	–
	+ CR	1.538	0.947–2.498	0.1
	+ NI	1.539	0.948–2.500	0.1
	+ welding	1.536	0.946–2.495	-0.1
	+ PAH	1.538	0.947–2.498	0.1
	+ ALHC	1.540	0.947–2.505	0.1
	+ ARHC	1.536	0.946–2.496	-0.1

NOTE: In the case of CHC, 1,1,1-trichloroethane, and Methylene chloride , adjustment for exposure to ALHC and ARHC is not justified. Though correlations were not too high, the apparent confounding effect was very high given the fact that exposure to aliphatic/alicyclic hydrocarbons or to aromatic hydrocarbons were not associated with Parkinson disease. This suggests strong collinearity of exposure between 1,1,1-trichloroethane, and Methylene chloride with ALHC and ARHC.

Appendix 1: Technical details of probabilistic bias analysis due to measurement error in exposure

We adopted the following notation: each ith subject in at j^{th} job for s^{th} solvent in t^{th} year was exposed to X_{ijst} exposure on average (this is also exposure accumulated in t^{th} year). This exposure was estimated as $E_{ijst}=P_{jst}\times L_{jst}$, i.e., all persons who worked in j^{th} job in t^{th} year were assigned the exposure value for s^{th} solvent by linkage of FINJEM to occupational histories captured in census records, documented as probability of exposure P at a level described by arithmetic mean L.[1] We will assume that the uncertainty in P_{jst} and L_{jst} is ignorable.

We simulated "true" exposure X_{ijst} given E_{ijst} assuming, like Burstyn et al.,[2] that X_{ijst} ~$(1-\omega_{jkt})+\omega_{jkt}X$, where ω_{jkt}~$Bern(P_{jst})$ (indicator variable for probability of being exposed when =1 and unexposed when =0), and X~$LogNorm(\mu_{ijst}, \sigma_{ijst}^2)$ (distribution of non-zero exposures). We have no information on σ_{ijst}^2 from FINJEM and assumed the default prior in distribution between-worker variance that appears to apply to wide range of jobs around the world, developed by Jones & Burstyn [3]: σ_{ijst}~Inverse-Gamma(shape=6.25, scale=4.80). The logarithmic mean of X is calculated as $\mu_{ijst}=\log(L_{jst})-0.5\sigma_{ijst}^2$.

Cumulative exposures were simulated as $C_{ijs} = \sum_{t=1}^{t=T} X_{ijst}$ over suitable disease induction period t=1 to t=T. In contrast, the naïve analysis estimated $C^*{}_{ijs} = \sum_{t=1}^{t=T} E_{ijst}$.

Imputed values of C_{ijs} were regressed on the outcome with control for covariates (see main text) via logistic regression to obtain log-odds ratios (θ) that account for *plausible systematic errors due to aggregation bias*. We incorporate random errors into these estimates by sampling log-odds ratios (log(ψ)) from distributions defined by point estimate resulting from adjustment for systematic error and its standard error, i.e., log(ψ)~Norm(θ, var(θ)). These were contrasted with conventional analyses that utilized exposure estimates of E_{ijst}. The simulations were repeated M=400 times to account for uncertainty in bias analysis and simulation intervals presented as 2.5^{th}, 50th, and 97.5^{th} percentiles.

Creating exposure categories using C^*_{ijs} makes study vulnerable to additional bias due to *differential misclassification due to dichotomization* (DDD) (see Singer at al.[4] for accessible explanation and illustration of a Bayesian methods to adjust for such bias). We quantify the impact of this method in probabilistic bias analysis. Specifically, we categorized C_{ijs} to gauge further the impact of this additional source of bias. The rest of analysis proceeded as for continuous cumulative exposure.

Implementation of these calculations in SAS is give below, via three programs: impute.sas, accumulate.sas, and analyze.sas.

***impute.sas;**

```
*Simulate presumed true exposures X as Bernoulli-lognormal mixture distribution by year;
*from FINJEM:
   *YYYY=1950 to 2008
   *P_EXP_YYYY = prevalence of exposure;
   *L_EXP_YYYY = average level of exposure of log-normal distribution;

/*set parameters of the simulations/imputations as a macro*/
%let NumImpute = 100; *number of imputations;
%let input= nomiss; *input dataset for bias analysis;
%let shape= 6.25; *a parameter of Inverse-Gamma distribution of log-variance of level of exposure from Method II of Jones & Burstyn (2017);
%let scale= 4.80; *b parameter of Inverse-Gamma distribution of log-variance of level of exposure from  Method II of Jones & Burstyn (2017);


data qba_CHC; set &input;
```

```
call streaminit(&NumImpute); /* generate reproducible random number stream from NumImpute*/
do ImputeID = 1 to &NumImpute;  /* create many imputed samples: each imputations defined by ImputeID */

/*impute level of exposure for every subject in every year in P_EXP_YYYY>0*/
/*impute variance:  sigma2, variance*/
/*show code only for 1950 and 2008 (first and last year) to make the illustration less repetitive; this step can be made more efficient with use of the ARRAYS*/

if          P_EXP_1950 >0 then sigma2_EXP_1950=1/RAND('GAMMA', &shape, 1/&scale);
/*insert years 1951 to 2007 */

if          P_EXP_2008 >0 then sigma2_EXP_2008=1/RAND('GAMMA', &shape, 1/&scale);
/*impute log-mean mu; if P_EXP_YYYY>0, else a missing value and error messages are generated due to log(0)*/
/*mu=log(AM)-0.5*VAR*/
if          P_EXP_1950 >0 then mu_EXP_1950=log(L_EXP_1950)-0.5*sigma2_EXP_1950;

/*insert years 1951 to 2007 */

if          P_EXP_2008 >0 then mu_EXP_2008=log(L_EXP_2008 )-0.5*sigma2_EXP_2008;

            /*assigned all those with P_EXP_YYYY=0 to X unexposed*/
if          P_EXP_1950 =0 then X_EXP_1950 =0;

/*insert years 1951 to 2007 */

if          P_EXP_2008 =0 then X_EXP_2008 =0;

/*when P_EXP_YYYY>0, then simulate individual indicator of omega_EXP_YYYY exposed =1 or unexposed=0*/
/*          if P>0 then omega=rand('Bernoulli', P);*/
if          P_EXP_1950 >0 then omega_EXP_1950=rand('Bernoulli', P_EXP_1950          );

/*insert years 1951 to 2007 */


if          P_EXP_2008 >0 then omega_EXP_2008=rand('Bernoulli', P_EXP_2008          );

/*for all those with P>0, if indicated as unexposed, X assign as unexposed*/
 /*if omega=0 then X&year = 0; /* 1-P subjects are unexposed, X is true exposure */
if          omega_EXP_1950 =0 then X_EXP_1950 =0;

/*insert years 1951 to 2007 */

if          omega_EXP_2008 =0 then X_EXP_2008 =0;

/* for all those with P>1, if indicated as exposed, impute exposure from the corresponding lognormal distribution*/
/*if omega=1 then X = rand("LogNormal", mu, log-StDev);*/ /* log-normal exposure for P subjects, X is true exposure */
If omega_EXP_1950=1 then X_EXP_1950=rand("LogNormal",mu_EXP_1950,sigma2_EXP_1950**0.5);

/*insert years 1951 to 2007 */


If omega_EXP_2008=1 then X_EXP_2008=rand("LogNormal",mu_EXP_2008,sigma2_EXP_2008**0.5);


output;
end;
```

```
run;

*accumulate.sas;
/*calculate cumulative exposures lagged by 5 years */

*input dataset is the one that is created by IMPUTE.sas;

data  qba_CHC_ce; set  qba_CHC;
ARRAY XCHC[59] X_EXP_1950-X_EXP_2008;
 * Making exposure as zero in calendar years after index_year-6 or after year when becomes 63.
        For example, for index year 2008 the last year contributing cumulative exposure is 2002
        or year whén 63, whichever comes first.
        Variable “vika” values correspond values 1 to 59,
        values of last_exposure years = actual calendar years.;
do i = (vika +1) to 59; XCHC[I]=0; LCHC[I]=0; END;

/*calculate imputed cumulative exposure, with LAG assumed in assigned values as per above conditions of VIKA*/
CE_EXP=       X_EXP_1950+  X_EXP_1951+  X_EXP_1952+  X_EXP_1953+  X_EXP_1954+  X_EXP_1955+
              X_EXP_1956+  X_EXP_1957+  X_EXP_1958+  X_EXP_1959+  X_EXP_1960+  X_EXP_1961+
              X_EXP_1962+  X_EXP_1963+  X_EXP_1964+  X_EXP_1965+  X_EXP_1966+  X_EXP_1967+
              X_EXP_1968+  X_EXP_1969+  X_EXP_1970+  X_EXP_1971+  X_EXP_1972+  X_EXP_1973+
              X_EXP_1974+  X_EXP_1975+  X_EXP_1976+  X_EXP_1977+  X_EXP_1978+  X_EXP_1979+
              X_EXP_1980+  X_EXP_1981+  X_EXP_1982+  X_EXP_1983+  X_EXP_1984+  X_EXP_1985+
              X_EXP_1986+  X_EXP_1987+  X_EXP_1988+  X_EXP_1989+  X_EXP_1990+  X_EXP_1991+
              X_EXP_1992+  X_EXP_1993+  X_EXP_1994+  X_EXP_1995+  X_EXP_1996+  X_EXP_1997+
              X_EXP_1998+  X_EXP_1999+  X_EXP_2000+  X_EXP_2001+  X_EXP_2002+  X_EXP_2003+
              X_EXP_2004+  X_EXP_2005+  X_EXP_2006+X_EXP_2007+X_EXP_2008;

run;

*analyze.sas;
*example given for cumulative continuous exposure.;

*imputed to account for aggregation bias inherent in FINJEM (SYSEMATIC ONLY);
*estimate logistic regression for each imputed dataset;
proc sort data = qba_CHC_ce;by ImputeID; run;
proc logistic data= qba_CHC_ce;
        class Sex(ref='1') SES_final(ref='3');
model case(event='1')= Sex birth_year SES_final personal_smoke ce_exp/ CLPARM=WALD;
ods output ParameterEstimates = Systematic;
by ImputeID;
run;

*retain in output only for cumulative exposure;
*account for SYSTEMATIC BIAS AND random error;
data Sys_Rand_CE_cont; set Systematic;
if Variable ne "CE_EXP" then delete;
EstimateSR=rand('Normal', Estimate, StdErr); /*CAPTURES BOTH SYSTEMATIC AND RANDOM ERRORS in LOG-OR, with
estimates log(Incidence Rate Ratio)*/
OR_SR=exp(EstimateSR);
keep ImputeID Variable Estimate StdErr EstimateSR OR_SR;
```

```
run;

*Summarize distribution of plausible effect estimates (IRRs) accounting for aggregation bias in exposure;
proc univariate data=Sys_Rand_CE_cont plot; var OR_SR; output out=percentiles pctlpre=P_ pctlpts=50 2.5 97.5; run;
proc print data=percentiles; run;

*end of SAS code;
```

References

1. Kauppinen T, Uuksulainen S, Saalo A, Makinen I, Pukkala E: **Use of the Finnish Information System on Occupational Exposure (FINJEM) in epidemiologic, surveillance, and other applications**. *Ann Occup Hyg* 2014, **58**(3):380-396.
2. Burstyn I, Lavoue J, Van TM: **Aggregation of exposure level and probability into a single metric in job-exposure matrices creates bias**. *AnnOccupHyg* 2012, **56**(9):1038-1050.
3. Jones RM, Burstyn I: **Bayesian Analysis of Occupational Exposure Data with Conjugate Priors**. *Ann Work Expo Health* 2017, **61**(5):504-514.
4. Singer AB, Daniele Fallin M, Burstyn I: **Bayesian Correction for Exposure Misclassification and Evolution of Evidence in Two Studies of the Association Between Maternal Occupational Exposure to Asthmagens and Risk of Autism Spectrum Disorder**. *Curr Environ Health Rep* 2018, **5**(3):338-350.
